# Alternative Operative Strategy in the Management of an Atrio-Esophageal Fistula

**DOI:** 10.7759/cureus.73471

**Published:** 2024-11-11

**Authors:** Sammy Shihadeh, Christoph A Stephenson-Moe, Paul Vesco, M. Blair Marshall

**Affiliations:** 1 Clinical Sciences, Florida State University College of Medicine, Tallahassee, USA; 2 Department of Cardiothoracic Surgery, Sarasota Memorial Health Care System, Sarasota, USA

**Keywords:** atrial fistula, atrial repair, esophageal fistula, esophageal repair, esophagectomy

## Abstract

An atrio-esophageal fistula is a rare sequela of ablation. Standard approaches are associated with a high mortality. Atrial ablation resulting in an atrio-esophageal fistula is associated with exceedingly high mortality. The treatment of an atrio-esophageal fistula is time-sensitive since complications such as sepsis and air emboli can arise from a delay in diagnosis, potentially worsening outcomes. We report an 82-year-old female patient presenting with right-sided hemi-paralysis, shortness of breath, productive cough, and intermittent fevers one month after an atrial radiofrequency ablation procedure. Our technique of fistula repair with division and ligation of the esophagus in this unstable patient, avoiding cardiopulmonary bypass, is discussed. Cardiopulmonary bypass requires systemic anticoagulation, and may not be suitable for a patient who presents with neurologic symptoms and evidence of embolic stroke on imaging due to the risk of hemorrhagic conversion. Her 22-day post-surgical management and course of recovery, which include a cervical esophagostomy, vocal cord paralysis, and right lower lobe pneumonia are also discussed. This off-bypass technique may be useful in some patients.

## Introduction

Atrial ablation for the treatment of atrial fibrillation, whether via cryoballoon or radiofrequency, can result in the rare complication of an atrio-esophageal fistula (AEF). Radiofrequency ablation leads to cellular necrosis by tissue heating up to 60 degrees Celsius. Cryoballoon therapy leads to cellular necrosis by tissue freezing using temperatures as low as negative 50 degrees Celsius [1]. AEF has an incidence of 0.03% and carries up to a 93% mortality risk following diagnosis [2]. The presentation of an AEF may vary, such as constitutional symptoms, odynophagia, hematemesis, and angina pectoris, although fever and neurological symptoms are the most common features with time to presentation ranging from two to four weeks up to two months [3]. The diagnosis of an AEF is critical and must not be overlooked, as further complications such as sepsis and infarctions from air emboli can arise due to a missed diagnosis, leading to additional morbidity/mortality.

Non-surgical management such as supportive care only or esophageal stenting is associated with extremely high mortality and is considered palliative only [4]. Fortunately, AEF can be surgically treated with fistula repair and/or esophagectomy. While there is no definitive standard for surgical repair of AEF, surgical approaches include median sternotomy with de-airing of the heart to prevent air-emboli (requiring cardiopulmonary bypass) or right or left thoracotomy depending on the location of the fistula (which may utilize bypass via femoral access if deemed necessary). Median sternotomy provides excellent exposure of the fistula; however, many of these patients are poor surgical candidates to begin with and may benefit from a less invasive approach. Also, cardiopulmonary bypass has a systemic inflammatory effect and may not be suitable for a patient who is septic on presentation. Potential techniques for fistula repair include placement of an interposition flap or, as in this patient, imbrication of the esophageal muscle layer with esophagectomy. Additionally, ligation and decompression of the cervical esophagus followed by spontaneous repair of the fistula have been described [5]. Subsequent to the emergency intervention, additional steps, whether surgical/medical management or palliative, should be discussed with the patient to promote their well-being and quality of life.

## Case presentation

An 82-year-old female patient with a history of right-sided lobectomy two years prior for lung adenocarcinoma and recurrent pleural effusion causing persistent dyspnea on exertion underwent evaluation and subsequent management of paroxysmal atrial fibrillation with radiofrequency ablation. Over the next several weeks, she developed increasing malaise and subsequent inability to tolerate an oral diet. Five weeks following the ablation procedure, she developed subjective fevers, chills/rigors, nausea, vomiting, and abdominal pain and presented to the emergency department. She had also been experiencing non-productive dry cough and dysphagia. She denied palpitations, chest pain, or dizziness.

**Figure 1:**
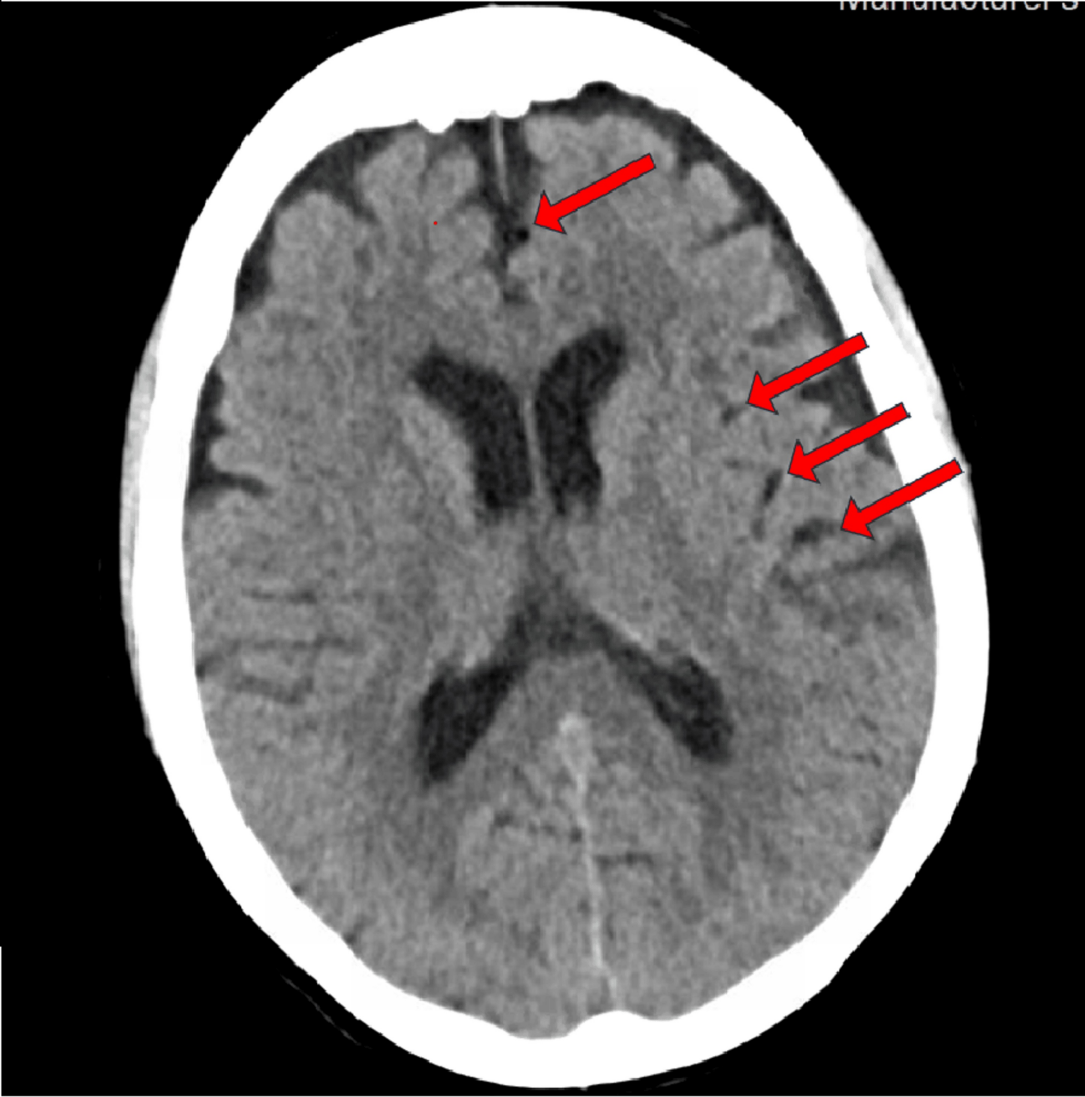
Computed tomography (CT) of the brain revealed air emboli present throughout the left cerebrum (indicated by the red arrows)

**Figure 2:**
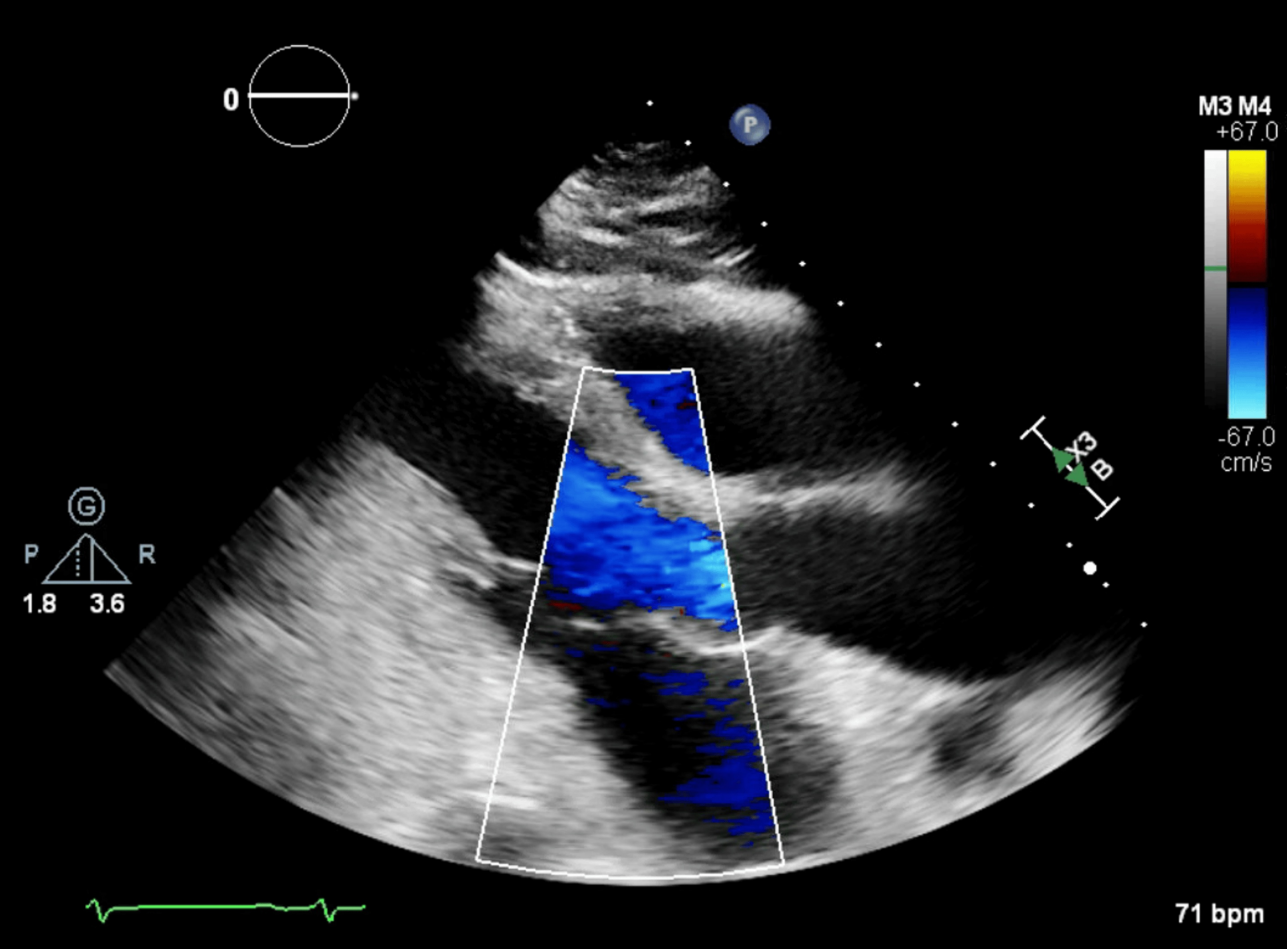
Echocardiography with Doppler flow demonstrating air in the left atrium

**Figure 3:**
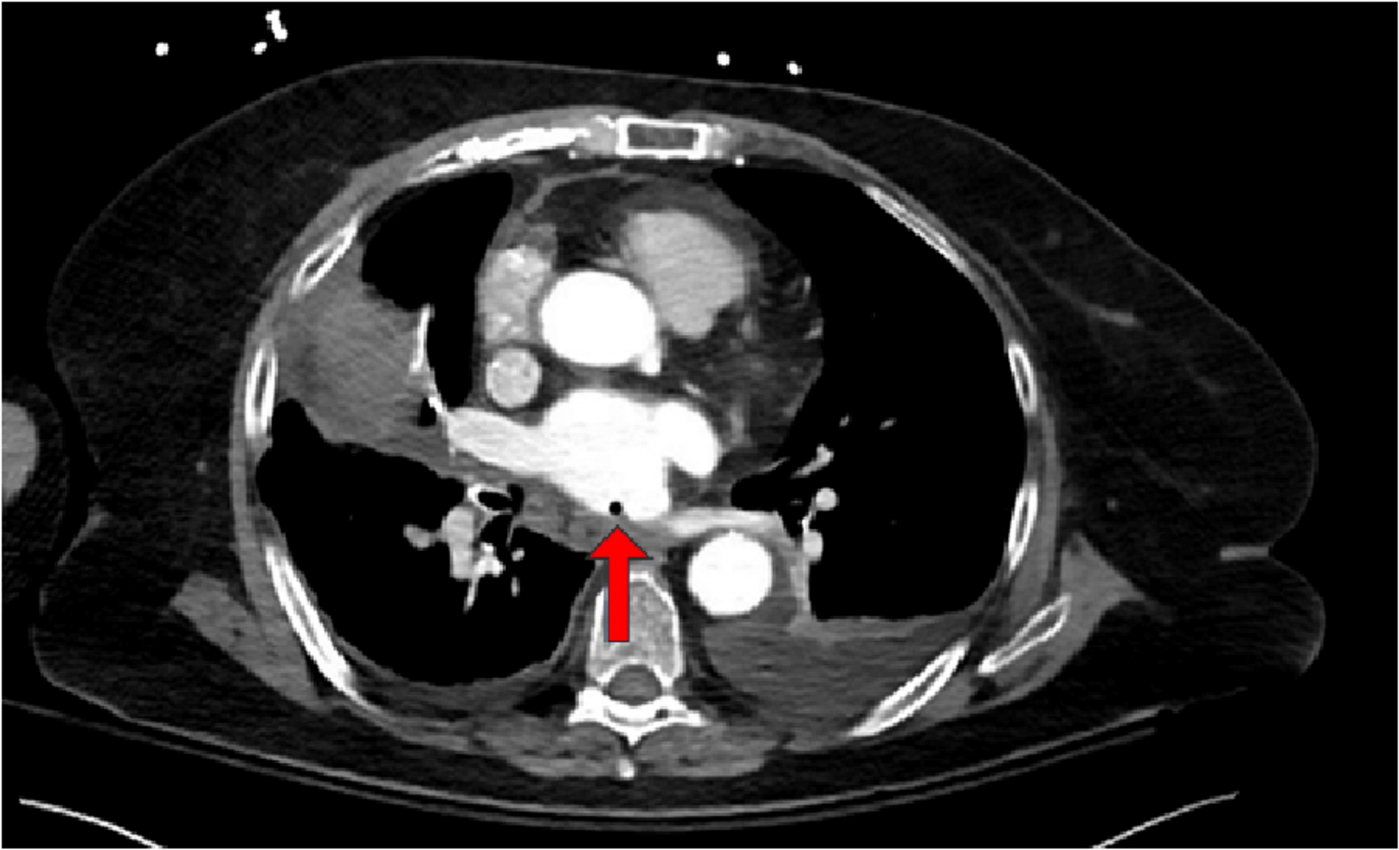
Transverse view computed tomography (CT) of the thorax demonstrating the fistula between the left atrium and the esophagus (indicated by the red arrow)

**Figure 4:**
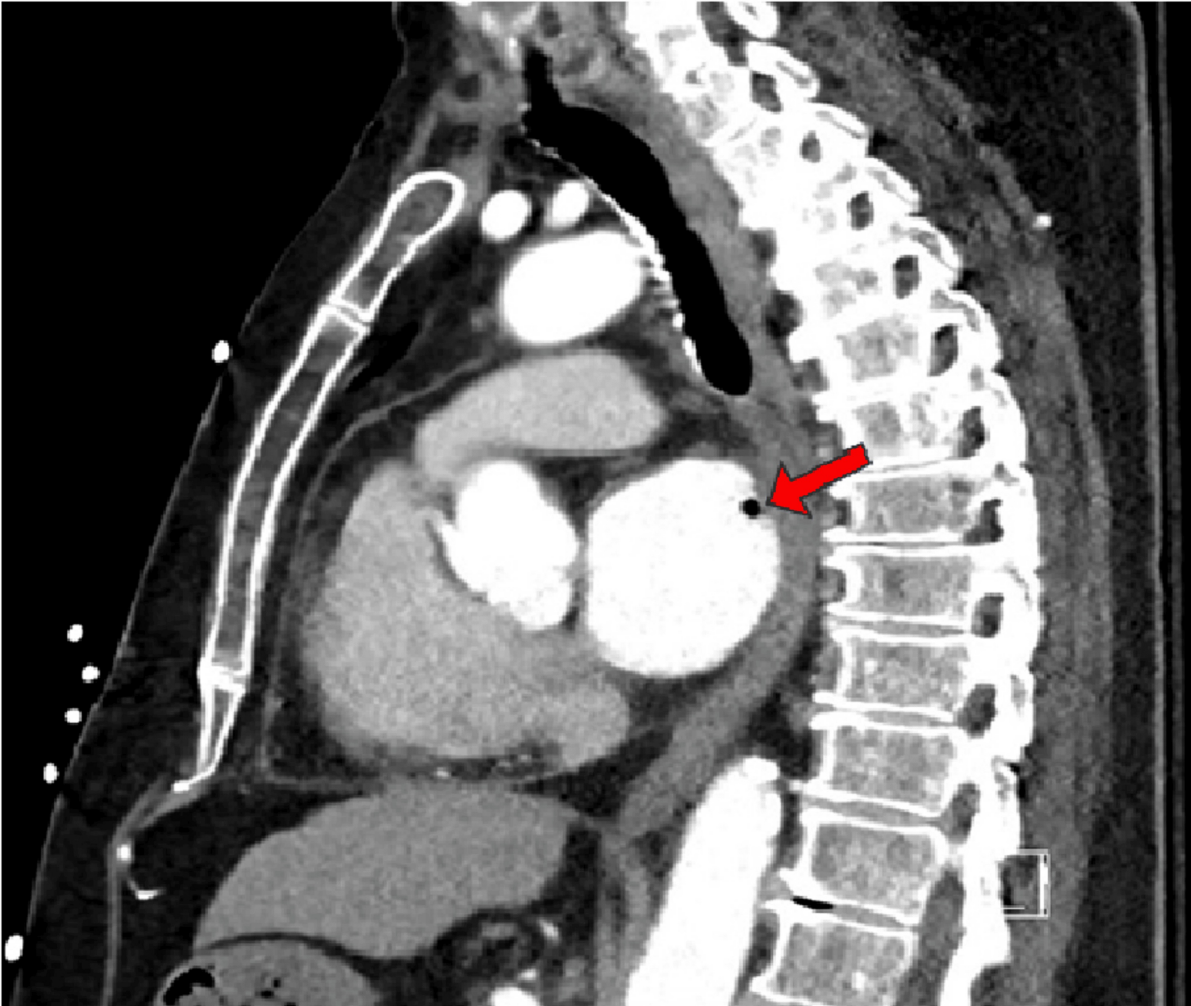
Sagittal view computed tomography (CT) of the thorax demonstrating the atrio-esophageal fistula (indicated by the red arrow)

She was admitted to her local hospital and was started on antibiotics. She was tachypneic on admission with a respiratory rate of 20 and had a white blood cell count of 12,100 cells/uL. Initial blood cultures grew *Gemella haemolysans*. She developed acute right-sided paralysis and non-contrast head computed tomography (CT) showed hypodensity in the parasagittal left frontal lobe indicating acute/subacute ischemia potentially secondary to multiple air emboli (Figure 1). The echocardiogram demonstrated air bubbles in the left atrium (Figure 2). Computed tomography angiography (CTA) pulmonary vein isolation demonstrated low attenuation focus within the posterior wall of the left atrium consistent with an air embolism (Figures 3, 4). She was urgently transferred for operative management.

Options for repair were considered and are often performed on cardiopulmonary bypass. However, in our experience, patients may develop fatal sepsis and risk hemorrhagic conversion of existing ischemic strokes. Considering her extremely poor state of health, presentation with sepsis and hemi-paralysis, albumin of 2.0 g/dL, prealbumin of 14.2 mg/dL, and a C-reactive protein level of 16 mg/dL (normal < 0.3 mg/dL) indicating elevated systemic inflammation, choices beyond the most common approach were considered. Cardiopulmonary bypass increases systemic inflammation and makes thrombus, and therefore, septic emboli formation more likely in a septic patient [6]. We decided that a division of the esophagus and an off-pump repair of the fistula would likely be associated with a lower risk of morbidity and mortality in this vulnerable patient.

Surgical techniques

In the operating room (OR), a percutaneous endoscopic gastrostomy (PEG) tube was placed with mild insufflation performed once past the lower esophageal sphincter. A left vertical muscle-sparing thoracotomy was performed, and the esophagus was circumferentially dissected above and below the fistula. Due to a previous right-sided lobectomy, the mediastinum was shifted into the right chest. This shift resulted in all of the structures in the chest being further away, past the spine in the right chest, and obscured by the aorta and the heart. To accommodate this anatomical variance, it was decided to slowly and meticulously dissect between the aorta and the heart to identify the esophagus, which was dissected circumferentially. The esophagus was then ensnared and divided above and below the fistula with a mechanical stapler. The heart was then rotated up further into the left chest and large Prolene mattress sutures (Ethicon, Inc., Raritan, New Jersey, United States) were used to pledget the esophageal muscle through the pericardium and left atrium.

The right side of the heart and the back wall of the left atrium were mobilized as well, and pledgeted interrupted mattress sutures were placed from the pericardial edge through-and-through the esophagus to the other side to obliterate the space between the esophageal lumen, the pericardium, and endocardium, thereby closing the fistula. Pledgets were used in particular due to their strength and durability in maintaining the closure of the fistula to promote healing due to the fistula’s large area. The esophageal mucosa was resected off the posterior pericardium with another stapler to prevent the formation of a mucocele in continuity with the atrium. Such mucoceles occur as a result of inflammatory processes (possibly due to surgical manipulation) and may enlarge over time, with the possible complication of obstruction. The chest was closed, and the patient was transferred to the intensive care unit.

When stable, the patient returned to the OR for a cervical esophagostomy. Postoperative complications included right lower lobe pneumonia, treated with antibiotics, and left vocal cord paresis, treated with direct injection with hyaluronic acid gel via microlaryngoscopy. These complications are suspected to be due to the procedure. The patient was discharged to rehabilitation on postoperative day 22. The patient has improved respiratory function including a louder voice volume, and the ability to cough. Ten months following discharge, she has some persistent right-sided weakness but is ambulating and preparing for reconstructive surgery of the esophagus for functional restoration.

## Discussion

Although catheter ablation is considered generally safe, it is not without risks of complications. Almost half of all patients with evidence of thermal esophageal injury from radiofrequency ablation typically recover without any additional morbidity [7]. Complication rates including hemorrhage and pericardial tamponade were found to not differ between patients who received radiofrequency ablation and cryoablation, as well as mortality rates, measuring at 0-2% among both groups [8]. The risk of AEF status post-atrial ablation depends on various factors such as thermal damage to the esophagus, non-brushing technique, and extended periods of compression for ablation, all of which may increase the risk of perforation from the left atrium into the esophagus. Atrial fibrillation is also associated with dilation and thinning of the atrium [9], making the structure vulnerable to heat damage during ablation. 

Bodziock et al. proposed some practices to help decrease the risk of AEF [10]. Such recommendations include conscious sedation instead of general anesthesia; a study demonstrated that only one patient (out of 25) receiving conscious sedation had evidence of esophageal damage versus 12 patients (out of 25) receiving general anesthesia displaying evidence of damage [11]. Irrigating the catheter tip is also proposed, as active cooling demonstrated decreased coagulation, lowered embolization risk, and more effective ablation of the left atrium wall, most efficacious with a low flow rate such as 2 mL/minute [12]. Shorter duration and higher energy of ablation (i.e., 90 W for four seconds) demonstrated transmural, continuous ablation versus longer duration but lower energy (i.e., 25 W for 20 seconds) showing incomplete transmural lesions with gaps in the area [13]. Intraoperative esophageal temperature monitoring, especially with an insulated probe, demonstrated decreased esophageal damage but still entails a risk of injury [14].

A mechanical positioning of the esophagus also promotes better outcomes, as the distance between the esophagus and the left atrium and the rise in esophageal temperature are inversely related [15]. A pharmacologic intervention such as proton pump inhibitors (PPIs) or H2 receptor antagonists (H2RAs) is also recommended to reduce gastric reflux, although currently there is not sufficient data about this practice. To avoid potentially causing/worsening an AEF, it is suggested to avoid any procedures that involve insufflation for at least five weeks after an ablation procedure due to the risk of AEF, and the diagnostic gold standard is to use CT with oral contrast studies, or nasogastric (NG) delivery of contrast for patients unable to swallow by raising the NG tube up to the suspected area [2]. Transthoracic echocardiography may be used to demonstrate air in the left atrium if clinical suspicion is high for AEF but is not a generally useful clinical tool in diagnosing AEF [16]; transesophageal echocardiography would be contraindicated in this circumstance. Additionally, other modalities to directly visualize the esophagus such as endoscopy are contraindicated due to potential worsening of the fistula and perforation. 

Alternative ablative strategies are potentially safer than conventional ablation and are currently in the experimental phase, which may help reduce the incidence of AEF in the future as these modalities continue to be developed and improved upon. For instance, a trial of pulsed-field ablation demonstrated successful pulmonary vein electrical isolation in all 475 patients, with only two patients developing pericardial effusion but recovering following treatment, and no cases of AEF or stroke were present [17]. A novel technology for atrial ablation using duty-cycled phased radiofrequency with an over-the-wire catheter was found to successfully isolate the pulmonary vein (PV) in 189 out of 190 patients with 8% of patients, demonstrating esophageal erythema and intramural bleeding based on a mean luminal esophageal temperature of 40.5 Celsius during ablation [18]. Another study demonstrated that remote magnetic navigation during ablation resulted in zero out of 3637 procedures resulting in AEF, while conventional ablation resulted in five out of 7016 procedures resulting in AEF [19].

The presence of severe infection and neurological conditions is a key presentation of an AEF, as in our patient, as this may be due to the air that is introduced from the esophagus into the left atrium during swallowing, which then may embolize from the left atrium into the systemic vasculature, and the mixing of gastric contents into the bloodstream, increasing the risk of sepsis. Additionally, surgical removal of parts of the esophagus may be considered if the extent and complications of the fistula are severe [20]. Due to our patient’s severe infection and the poor condition of the tissue, esophagectomy was performed to prevent the spread of infection and facilitate fistula repair.

AEF repair on cardiopulmonary bypass has been described, but these methods may be associated with an increased risk of mortality from disseminated sepsis due to bacterial contamination from the fistula [21]. Additionally, because of the patient’s ischemic stroke from air emboli, cardiopulmonary bypass was contraindicated as it requires heparinization, which might have converted the stroke from ischemic to hemorrhagic. For these reasons, it was decided not to perform the procedure on cardiopulmonary bypass. Because of our patient’s elderly age, malnutrition, sepsis, and paralysis, we opted for esophagectomy division and diversion as the initial means of management. Although primary esophageal repair without cardiopulmonary bypass has been described, given the chronicity of our patient’s pathology, and the mass-like phlegmon involving the atrium and the esophagus, we did not think that this approach was feasible [22]. Had the patient presented without her other symptoms in association with the AEF, operating on cardiopulmonary bypass may have been considered.

## Conclusions

Atrial ablation can present with the adverse event of AEF. Sepsis compounded with stroke and malnutrition are co-morbidities often leading to increased mortality. Strategies for repair should be individualized for each patient and when the patient’s risk of mortality is extremely high, esophagectomy may be the least morbid option. Although atrial fibrillation can be treated safely with ablation most of the time, there is a small risk of the formation of an AEF due to variables such as thermal damage to the esophagus and the atrium. The onset of symptoms is usually delayed and therefore when a patient appears septic with neurological deficits, chest pain, and/or odynophagia after having had an ablation procedure within the past month or so, clinical suspicion of AEF should remain high. Rapid diagnosis with contrast-based CT is optimal as it can readily demonstrate a fistula in a timely manner. Endoscopy should also be avoided due to potentially worsening the fistula and introducing air emboli. The choice of esophagectomy, particularly in high-risk cases, should consider both immediate risks and potential long-term outcomes for the patient, although esophagectomy may offer a survival advantage in unstable patients. Long-term follow-up with the patient is also important to ensure the resolution of the fistula, and future interventions may also be discussed.
